# Bounding basis reduction properties

**DOI:** 10.1007/s10623-016-0273-9

**Published:** 2016-09-15

**Authors:** Arnold Neumaier

**Affiliations:** Fakultät für Mathematik, Universität Wien, Oskar-Morgenstern-Platz 1, 1090 Vienna, Austria

**Keywords:** Basis reduction, Lattices, BKZ algorithm, LLL algorithm, 11H55, 11Y16

## Abstract

The paper describes improved analysis techniques for basis reduction that allow one to prove strong complexity bounds and reduced basis guarantees for traditional reduction algorithms and some of their variants. This is achieved by a careful exploitation of the linear equations and inequalities relating various bit sizes before and after one or more reduction steps.

## Introduction

Reduction algorithms for bases of lattices play an important role in algorithmic number theory, cryptography, and integer programming; see, e.g., Nguyen and Vallée [25] and the references given there.

Most existing basis reduction algorithms (see, e.g., [9, 11, 12, 14, 18–21, 24, 30–33, 36, 39]) proceed by progressively updating the basis. These updates are derived from a Gram–Schmidt orthogonalization or QR factorization, equivalent to the Cholesky factorization of the Gram matrix. Analysing these updates, proving the polynomial complexity of the resulting algorithms, and proving bounds on the quality of the final reduced basis are nontrivial tasks.

Early work by Lagrange [17] in two dimension and by Hermite [15] in general dimensions culminated in the LLL algorithm by Lenstra et al. [18], which produces in polynomial time in the bit size of an input basis a reduced basis whose basis vectors have bit sizes bounded by a fixed multiple of the dimension. Many variants of the original LLL algorithm exist, so we have in fact a whole class of LLL algorithms. These are characterized by working at any time on 2-dimensional projected subspaces only, and are sufficient for many applications.

Stronger basis reduction algorithms are needed in case the LLL reduced basis is still not short enough. Korkine and Zolotareff [16] introduced what are today (after them and Hermite) called HKZ reduced bases with excellent theoretical properties. But their computation is feasible at present only for low-dimensional lattices (up to dimensions around 75). Thus one uses in practice block algorithms; they apply strong and expensive reduction techniques on low-dimensional projected subspaces only. Currently the best practical algorithms are the BKZ algorithm (Schnorr and Euchner [32]) and the recent self-dual SDBKZ variant by Micciancio and Walter [21] (called DBKZ there). On the other hand, the best theoretical guarantees for block algorithms are provided by the (at currently practical block sizes apparently inferior) slide reduction algorithm of Gama and Nguyen [11].

In this paper, the approaches of Hanrot et al. [14] (used also in Micciancio and Walter [21]), Schnorr [31], and Gama and Nguyen [11] for the asymptotic worst-case analysis of LLL, BKZ, and SDBKZ are improved. The first improvement replaces the complicated dynamical system arguments of [14] by simpler and sharper induction arguments on a bound on the bit sizes. The second improvement is an analysis of a greedy variant of LLL that is quasilinear in the bit sizes and has a guarantee on the approximation factor. Based on the techniques of the present paper, Neumaier and Stehlé [23] present an analysis of another, recursive variant of LLL that gives the asymptotically fastest method so far.

To make the paper self-contained, we present the relevant background on lattices and basis reduction in a novel way, namely in terms of bit sizes and linear inequalities relating these. This form was inspired by Hanrot et al. [14] who reduced most of the complexity analysis of basis reduction methods to a study of linear equations and inequalities. Before their work, this underlying linear structure was invisible since the analysis was—with the single exception of Schönhage [33, Lemma 4.1]—always done in a multiplicative way.

## Basic notions

This section provides basic definitions together with a collection of mostly well-known results put together in a form useful for the subsequent development. In view of further applications to be reported elsewhere, some of the results are presented in slightly greater generality than needed in this paper.

### The bit profile

A **lattice** of **dimension** *n* is a nonempty subset $\mathbb {L}$ of the space $\mathbb {R}^m$ of *m*-dimensional column vectors with real entries (for some *m*) that is closed under subtraction and has a **basis**, i.e., a matrix $B=[b_1,\ldots ,b_n]$ with *n* linearly independent columns $b_i$ generating $\mathbb {L}$. Given the basis,

1 $$\begin{aligned} \mathbb {L}=\{Bz\mid z\in \mathbb {Z}^n\}; \end{aligned}$$

conversely, if $B\in \mathbb {R}^{m\times n}$ has rank *n* then (1) defines a lattice with basis *B*. The matrix

$$\begin{aligned} G=B^TB \end{aligned}$$

is called the **Gram matrix** of the basis. We call the submatrices $B_{i:k}:=[b_i,\ldots ,b_k]$ the **subbases** of *B*; its Gram matrices $G_{1:i}:=B_{1:i}^TB_{1:i}$ are the leading submatrices of *G*. The **bit profile** of *B* is the sequence $g_0,\ldots ,g_n$ of **determinant bit sizes** 1

2 $$\begin{aligned} g_i:=\lg \det G_{1:i} \end{aligned}$$

of the leading subdeterminants

$$\begin{aligned} d_i:=\det G_{1:i} \end{aligned}$$

of the Gram matrix. Here

$$\begin{aligned} \lg x=\ln x/\ln 2 \end{aligned}$$

denotes binary logarithms, and the determinant of a $0\times 0$ matrix is taken to be 1, so that $g_0=0$.

The **dual lattice** $\mathbb {L}^\dagger$ consists of the linear combinations *y* of lattice vectors such that $y^Tx$ is integral for all $x\in \mathbb {L}$. If *B* is a basis of $\mathbb {L}$ then, with the permutation matrix *J* defined by $(Jx)_i:=x_{n+1-i}$, the reversed **dual basis** $B^\dagger =BG^{-1}J$ is a basis of $B^\dagger$ with Gram matrix $G^\dagger =JG^{-1}J$. Since the leading subdeterminants of $G^\dagger$ satisfy $\det G^\dagger _{1:i}=\det G_{1:n-i}/\det G$, its determinant bit sizes are given by

3 $$\begin{aligned} g_i^\dagger =g_{n-i}-g_n. \end{aligned}$$

The $k\hbox {th}$ **block** $B^{k:k+s-1}$ of size $s\le n$ ($k=1,\ldots ,n+1-s$) of a basis *B* of dimension *n* is the projected basis of dimension *s* obtained by orthogonalizing the subbasis $B_{k:k+s-1}$ against the basis vectors in $B_{1:k-1}$. (For $k=1$, we have $B^{1:s}=B_{1:s}$.) The corresponding determinant bit sizes are the numbers

4 $$\begin{aligned} g_i^{(k)}:=g_{k+i-1}-g_{k-1}~~~ (i=1,\ldots ,s). \end{aligned}$$

It is customary to denote the first basis vector of the blocks $B^{i:n}$ by $b_i^*$; then

5 $$\begin{aligned} q_i:=\Vert b_i^*\Vert ^2=\frac{d_i}{d_{i-1}}=2^{e_i} \end{aligned}$$

with the **projected bit sizes**

6 $$\begin{aligned} e_i:=g_i-g_{i-1}=\lg \Vert b_i^*\Vert ^2 ~~~\text{ for } i=1,\ldots ,n. \end{aligned}$$

In particular,

$$\begin{aligned} \prod _{i=1}^n \Vert b_i^*\Vert =(\det G)^{1/2}. \end{aligned}$$

We also use the **normalized projected bit sizes**

7 $$\begin{aligned} a_i:=e_1-e_i=g_1+g_{i-1}-g_i. \end{aligned}$$

They are invariant under rescaling of the basis by a constant factor, and we have

$$\begin{aligned} a_1=0. \end{aligned}$$

From the $a_i$ and $g_1$ we can recover

$$\begin{aligned} e_i=g_1-a_i, \end{aligned}$$

$$\begin{aligned} g_i=ig_1-a_1-\ldots -a_i. \end{aligned}$$

We call

8 $$\begin{aligned} \sigma (g):=\max _{\ell >j}\, (a_\ell -a_j) \end{aligned}$$

the **spread** of the basis.

#### Proposition 2.1

For $$0\le i\le k\le n$$,

9 $$\begin{aligned} \frac{g_i}{i}-\frac{g_k}{k} \le \frac{k-i}{k}\max _{j<\ell \le k}(a_\ell -a_j) \le \sigma (g). \end{aligned}$$

#### Proof

We have

$$\begin{aligned} kg_i-ig_k= & {} i(a_1+\ldots +a_k)-k(a_1+\ldots +a_i) =\displaystyle \sum _{j=1:i,\ell =i+1:k} (a_\ell -a_j)\\\le & {} i(k-i)\displaystyle \max _{j<\ell \le k}(a_\ell -a_j) \le ik\sigma (g). \end{aligned}$$

Division by *ik* establishes the claim. $\square$

Daudé and Vallée [9] show that for a basis *B* of dimension *n* with random entries of sufficiently large bit sizes and under reasonable assumptions on the distribution, the spread $\sigma (g)$ has an expected value of $<5+\ln n$. This kind of random basis is relevant in signal processing. On the other hand, unreduced lattice bases from cryptography often—e.g., the Coppersmith lattices for polynomial factorization [8]—have a spread of order $n^2$. LLL-reduced lattice bases have $\sigma (g)=O(n)$; cf. (37) below.

**Fig. 1:**
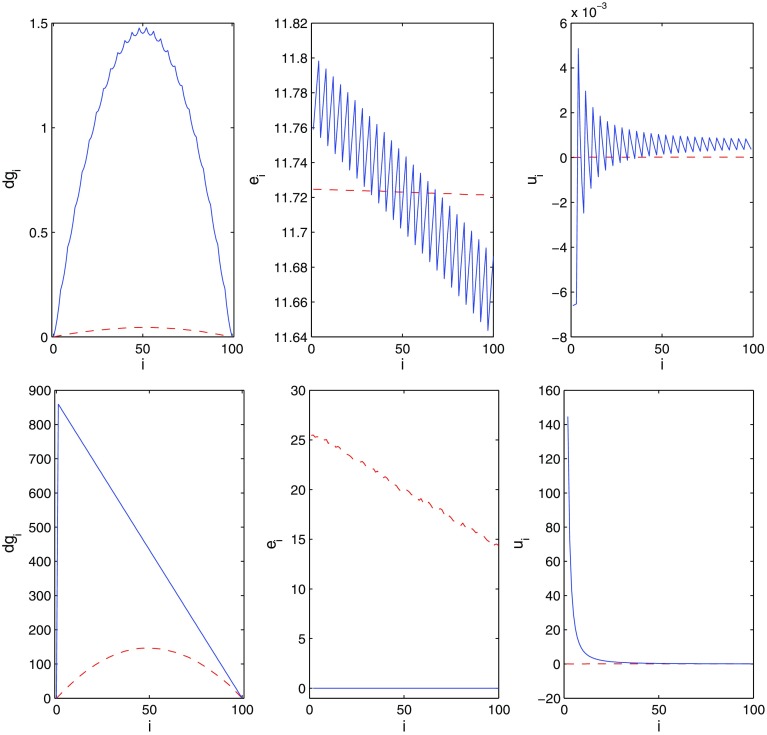
From *left* to *right*: differences $dg_i, e_i, u_i$ of two bit profiles. *Blue solid line* input, *red dashed line* LLL output. *Top* Coppersmith lattice, *bottom* SVP lattice (Color figure online)

For graphical display, the bit profile $g_i$ usually looks unconspicuous; the interesting information is in various differences. Figure 1 displays $dg_i=g_i-\displaystyle \frac{i}{n}g_n, e_i$, and $u_i:=\displaystyle \frac{g_i}{i}-\frac{g_{i+1}}{i+1}$, cf. (10), for the input basis and an LLL-reduced basis computed by fplll [37], for an example from the Coppersmith method and an example from a shortest vector problem from the SVP challenge page [4]. In the second problem, the entries $e_1\approx 868$ and $u_1\approx 434$ are not shown.

By definition of the **Rankin invariants** $\gamma _{ni}$ of Gama et al. [10], every lattice has a basis for which the inequalities

10 $$\begin{aligned} \frac{g_i}{i}-\frac{g_k}{k}\le \frac{\log \gamma _{ki}}{i} \end{aligned}$$

hold. The most important special case of the Rankin invariants is the **Hermite constant** $\gamma _n=\gamma _{n1}$, the largest possible value of $\displaystyle \min _{0\ne z\in \mathbb {Z}^n} \Vert Bz\Vert ^2$ for a matrix *B* of rank *n* with $\det (B^TB)=1$. What is known about Hermite constants and other Rankin invariants is reviewed in Appendix; here we just note that $\gamma _1=1, \gamma _2=2/\sqrt{3}$, and

$$\begin{aligned} \frac{1}{17.08}<\frac{\gamma _n-1}{n-1}\le \frac{1}{7}. \end{aligned}$$

In the following we shall need the constants

11 $$\begin{aligned} \Gamma _n:=\lg \gamma _n,\,\mu _n:=\frac{\Gamma _n}{n-1}. \end{aligned}$$

### Primal and dual reduction

The goal of **basis reduction** is to construct from a given basis *B* of a lattice $\mathbb {L}$ another basis consisting of shorter vectors. Various criteria for reducedness quantify the extent to which this is achieved. We say that a basis *B* is **size reduced** if

$$\begin{aligned} |(b_i^*)^Tb_k^*| \le \frac{1}{2}\Vert b_k^*\Vert ^2 ~~~\text{ for } i>k. \end{aligned}$$

A basis *B* is **primal reduced** if the length of the first basis vector $b_1$ is a shortest nonzero lattice vector. Every leading block $B^{1:i}$ is then also primal reduced. A basis *B* is **dual reduced** if the reversed dual basis is primal reduced. Every trailing block $B^{i:n}$ is then also dual reduced.

The process of **size reduction** (resp. **primal reduction**, **dual reduction**) replaces an arbitrary basis by one that is size reduced (resp. primal reduced, dual reduced). Size reduction is achievable by subtracting for $i=2,\ldots ,n$ from $b_i$ an appropriate integral linear combination of $b_1,\ldots ,b_{i-1}$. For block size $s=2$, primal and dual reduction are equivalent. An efficient algorithm for performing the reduction of a block of size $s=2$ goes back to the 18th century [17]. We therefore call this process **Lagrange reduction**. For primal or dual reduction of block size $s>2$, one must first solve a shortest vector problem, then transform the basis accordingly; see Micciancio and Walter [21, Sect. 7] for economical procedures. The **shortest vector problem** (**SVP**) is the problem to find, given a basis *B*, a shortest nonzero vector *z* of the lattice $\mathbb {L}$ spanned by *B*, thus achieving the **minimum length**

$$\begin{aligned} \lambda _1(B):=\min _{0\ne z\in \mathbb {L}}\Vert z\Vert \end{aligned}$$

The following result (trivial for $m=1$) is implicit in Gama and Nguyen [11, proof of Theorem 1], who strengthened an observation of Lenstra et al. [18, proof of Proposition 1.11] (the case $m=n$, where the hypothesis is trivially satisfied) in order to obtain an improved bound for the approximation factor of slide reduction; cf. (27) below. Related results are in Pataki and Tural [27].

#### Proposition 2.2

If $B^{m:n}$ is primal reduced then

12 $$\begin{aligned} \min _{k=1:m}e_k \le \lg \lambda _1(B)^2 \le e_1. \end{aligned}$$

In particular, this always holds for $m=n$.

#### Proof

We may write a shortest nonzero vector *b* as an integral linear combination

$$\begin{aligned} b=Bz=\sum _i z_i b_i ~~~(z\in \mathbb {Z}^n) \end{aligned}$$

of the basis vectors. Let *k* be the largest index with $z_k\ne 0$. If $k<m$ then

$$\begin{aligned} \lambda _1(B)=\Vert b\Vert \ge |z_k|\,\Vert b_k^*\Vert \ge \Vert b_k^*\Vert =\sqrt{e_k}, \end{aligned}$$

while if $i\ge m$ then

$$\begin{aligned} \lambda _1(B)=\Vert b\Vert \ge \Big \Vert \sum _{i\ge m} z_i b_i\Big \Vert \ge \lambda _1(B^{m:n}) =\Vert b_m^*\Vert =\sqrt{e_m}. \end{aligned}$$

$$\square $$

#### Proposition 2.3

(i) Upon primal reduction of a block $B^{k:k+s-1}$, the modified bit profile $g_i'$ of *B* satisfies $g_i'=g_i$ unless $k\le i\le k+s-2$, and we have
  13 $$\begin{aligned}&\displaystyle \min _{\ell =0:s-1}e_{k+\ell }\le e_k'\le e_k, \end{aligned}$$
  14 $$\begin{aligned}&\displaystyle 0\le g_k-g_k'\le \max _{\ell =0:s-1}(a_{k+\ell }-a_k). \end{aligned}$$

(ii) Upon dual reduction of a block $B^{k-s+2:k+1}$, the modified bit profile $g_i'$ of *B* satisfies $g_i'=g_i$ unless $k\le i\le k+s-2$, and we have
  15 $$\begin{aligned}&\displaystyle e_{k+1}\le e_{k+1}'\le \max _{\ell =1:s-1}e_{k+1-\ell }, \end{aligned}$$
  16 $$\begin{aligned}&\displaystyle 0\le g_k-g_k'\le \max _{\ell =1:s-1}(a_{k+1}-a_{k+1-\ell }). \end{aligned}$$

#### Proof

(i) We apply (12) to the primal reduction of the block $B^{k:k+s-1}$ and find (13). As a consequence,
  $$\begin{aligned} 0\le e_k-e_k' \le e_k-\min _{\ell =0:s-1}e_{k+\ell } =\max _{\ell =1:s-1}(e_k-e_{k+\ell }) =\max _{\ell =1:s-1}(a_{k+\ell }-a_k). \end{aligned}$$
  (14) follows since $$g_{k-s+1}-g_{k-s+1}'=e_{k-s+1}-e_{k-s+1}'$$.

(ii) follows from (i) applied to the dual basis with $n-k$ in place of *k*, using (3) which implies $e_i^\dagger =-e_{n+1-i}$.

$$\square $$

Rescaling an arbitrary lattice basis *B* to one whose Gram matrix has determinant 1, the definition of the Hermite constants gives

17 $$\begin{aligned} \gamma (B):=\frac{\lambda _1(B)^2}{d_n^{1/n}}\le \gamma _n. \end{aligned}$$

Clearly, $\gamma (B)$ is basis-independent and depends only on the lattice generated by *B*. For a basis *B* of a random lattice (drawn uniformly according to the Haar measure; cf. Goldstein and Meyer [13]), Rogers [28] (see also Södergren [35]) proved that in the limit $n\rightarrow \infty$, the probability that $\gamma (B)>\gamma$ is given by

$$\begin{aligned} \Pr (\gamma (B)>\gamma )=e^{-\frac{1}{2}\pi _n \gamma ^{n/2}} ~~~\text{ for } \gamma \ge 0, \end{aligned}$$

where

$$\begin{aligned} \pi _n:=\frac{\pi ^{n/2}}{\Gamma (n/2+1)} \end{aligned}$$

denotes the volume of the unit ball in $\mathbb {R}^n$. In particular, the median of $\gamma (B)$ is

18 $$\begin{aligned} \gamma _n^*=\Big (\frac{2\log 2}{\pi _n}\Big )^{2/n} =\pi ^{-1}\Big (2\log 2\cdot \Gamma (n/2+1)\Big )^{2/n}, \end{aligned}$$

and the median of $(n-1)/(\gamma (B)-1)$ is

$$\begin{aligned} \frac{n-1}{\gamma _n^*-1} \approx 2e\pi \Big (1+\frac{2}{n}\log n\Big ) \end{aligned}$$

with an error of $O(n^{-1})$. This is monotone decreasing for $n\ge 12$ and converges very slowly to $2e\pi \approx 17.094$, and is approximately 20 for *n* between 60 and 75. The so-called Gaussian heuristic—obtained by a more informal sphere packing argument—assumes the slightly simpler formula $\gamma (B)\approx \pi ^{-1}\Gamma (1+n/2)^{2/n}$ with the same asymptotics. Unless *n* is large, both formulas give values that are too small. It may be better to use instead the heuristic

19 $$\begin{aligned} \gamma (B)\approx 1+\frac{n-1}{2e\pi (1+\frac{2}{n}\log n)} ~~~\text{ or } \text{ even } \gamma (B)\approx 1+\frac{n-1}{20}. \end{aligned}$$

#### Proposition 2.4

(i) If the block $B^{k:k+s-1}$ is primal reduced then
  20 $$\begin{aligned} g_k-g_{k-1}-\frac{1}{s}(g_{k+s-1}-g_{k-1})\le \Gamma _s. \end{aligned}$$

(ii) If the block $B^{k-s+2:k+1}$ is dual reduced then
  21 $$\begin{aligned} g_k-g_{k+1}-\frac{1}{s}(g_{k-s+1}-g_{k+1})\le \Gamma _s. \end{aligned}$$

#### Proof

Upon scaling the subbasis $B_{1:s}$, the definition of the Hermite constants implies that $\det G_{1:1}\le \gamma _s(\det G_{1:s})^{1/s}$. Take logarithms to get

22 $$\begin{aligned} g_1-\frac{g_s}{s} \le \Gamma _s. \end{aligned}$$

(22) applied to the block $B^{k:k+s-1}$ gives (i). (ii) follows by applying (22) to the dual of the block $B^{k-s+2:k+1}$ with bit sizes derived from (3). $\square$

Given a basis of dimension $n+1$ and determinant 1 (so that $g_0=g_{n+1}=0$), we may alternate primal reduction of $B^{1:n}$ and dual reduction of $B^{2:n+1}$ until $g_1$ no longer decreases. This is a finite process as there are only finitely many vectors in the lattice shorter than any given vector. The resulting basis satisfies (20) for $i=1,s=n$ and (21) for $i=s=n$,

$$\begin{aligned} g_1-\frac{g_n}{n} \le \Gamma _n,~~~ g_n-\frac{g_1}{n}\le \Gamma _n. \end{aligned}$$

Multiplying the first inequality by *n* and adding the second inequality gives after division by $n^2-1$ the bound $\displaystyle \frac{g_1}{n}\le \frac{\Gamma _n}{n-1}=\mu _n$. Since $\Gamma _{n+1}$ is the supremum of the left hand side over all bases of dimension $n+1$ and determinant 1, we find

23 $$\begin{aligned} \mu _{n+1}\le \mu _n~~~\text{ for } n\ge 2, \end{aligned}$$

which is Mordell’s inequality (Mordell [22]).

### Basis quality

There are a number of indicators that quantify the quality of a reduced basis. Gama and Nguyen [12] define the **Hermite factor**

$$\begin{aligned} H(B):=\frac{\Vert b_1\Vert }{(\det G)^{1/(2n)}} \end{aligned}$$

and the **root Hermite factor**

$$\begin{aligned} R(B):=H(B)^{1/n} \end{aligned}$$

of a basis *B*. (Using the $(n-1)$st root would be more appropriate.) Expressed in terms of the **Hermite exponent**

24 $$\begin{aligned} h(g):=\frac{ng_1-g_n}{n(n-1)}=\frac{a_1+\ldots +a_n}{n(n-1)} \end{aligned}$$

we have

25 $$\begin{aligned} H(B)=2^{\frac{n-1}{2}h(g)},~~~R(B)=2^{\frac{n-1}{2n}h(g)}. \end{aligned}$$

If the basis is primal reduced then (17) gives $H(B)=\sqrt{\gamma (B)}$, hence $h(g)\le \mu _n$ by (22). By definition of the Hermite constants, there are lattices of every dimension *n* for which no basis can have a better Hermite exponent.

The **approximation factor** (or **length defect**) of a basis *B* is the quotient

$$\begin{aligned} A(B):=\frac{\Vert b_1\Vert }{\lambda _1(B)}\le 2^{\frac{n-1}{2}a(g)}, \end{aligned}$$

where

26 $$\begin{aligned} a(g):=\frac{1}{n-1}\max _i a_i \end{aligned}$$

denotes the **approximation exponent**. If $B^{m:n}$ is primal reduced then Proposition 2.2 implies the slightly stronger bound

27 $$\begin{aligned} A(B)\le 2^{\frac{m}{2}\widetilde{a}_m(g)} \end{aligned}$$

with the modified exponent

$$\begin{aligned} \widetilde{a}_m(g):=\frac{1}{m}\max _{i\le m} a_i. \end{aligned}$$

In a signal processing context,

$$\begin{aligned} \pi (B):=\frac{\lambda _1(B)^2}{\displaystyle \min _i q_i} \le \frac{q_1}{\displaystyle \min _i q_i} =2^{(n-1)a(g)} \end{aligned}$$

is called the **SIC proximity factor**. The **effective dimension**

28 $$\begin{aligned} n_{{{\text{ eff }}}}:=\max \{k\mid a_k>0\}=\max \{k\mid {e_k}<{e_1}\} =\max \{k\mid \Vert b_k^*\Vert <\Vert b_1^*\Vert \} \end{aligned}$$

is the smallest value of *m* for which (12) implies that the basis vectors with $k>m$ cannot contribute to a vector shorter than the first basis vector. We call the vectors $b_i$ with $i\le m$ the **effective basis vectors**, the Hermite exponent $h(g_{1:m})$ the **effective Hermite exponent**, and the approximation exponent $a(g_{1:m})$ the **effective approximation exponent**. By definition, we still have

29 $$\begin{aligned} a(B)\le 2^{\frac{n-1}{2}a(g_{1:m})}, \end{aligned}$$

Of interest is also the **normalized spread**

30 $$\begin{aligned} c(g):=\frac{\sigma (g)}{n-1}; \end{aligned}$$

cf. (8). Note that

31 $$\begin{aligned} h(g)\le a(g)\le c(g); \end{aligned}$$

thus proving a small bound on *c*(*g*) is the strongest form of reduction guarantee. If *B* is size reduced then (using QR factors)

$$\begin{aligned} \frac{\Vert b_i\Vert ^2}{\Vert b_i^*\Vert ^2} =\frac{1}{q_i}\sum _{j=1}^{i}(b_i^*)^Tb_j^* \le 1+\frac{1}{4q_i}\sum _{j=1}^{i-1}q_j=\kappa _i, \end{aligned}$$

where

$$\begin{aligned} \kappa _i:=1+\frac{1}{4}\sum _{j=1}^{i-1}2^{e_j-e_i} =1+\frac{1}{4}\sum _{j=1}^{i-1}2^{a_i-a_j} \le 1+\frac{i-1}{4}2^{(n-1)c(g)}< 2^{(n-1)c(g)}\,i, \end{aligned}$$

The **orthogonality defect** is the number

$$\begin{aligned} \mathop {\mathrm{od}}(B):=(\det G)^{-1/2}\prod _{i=1}^n \Vert b_i\Vert =\prod _{i=1}^n \frac{\Vert b_i\Vert }{\Vert b_i^*\Vert } \le \Big (\prod _{i=2}^n \kappa _i\Big )^{1/2} < 2^{\frac{1}{2}\big ((n-1)^2c(g)+\lg n!\big )}. \end{aligned}$$

Since $\Vert b_i\Vert \ge \Vert b_i^*\Vert$ we see that $\mathop {\mathrm{od}}(B)\ge 1$, which is **Hadamard’s inequality**. Finally, we may also consider the **mean slope**

$$\begin{aligned} \frac{e_1-e_n}{n-1}=\frac{a_n}{n-1} \end{aligned}$$

of the sequence $e_1,\ldots ,e_n$, which is a mean curvature of the bit profile.

## Block reduction

Limiting the size of the quality measures discussed in Sect. 2.3 is a key task to be achieved by basis reduction. In particular, one would like to have small, dimension-independent bounds for the numbers in (31).

The most frequently used algorithms for basis reduction are variants of the LLL algorithm of Lenstra et al. [18] and the BKZ algorithm of Schnorr and Euchner [32]. On the other hand, when primal or dual reductions are done for blocks of size at most *s* only (with fixed *s*), the currently best guarantees for the reduced basis are given—when *s* divides the dimension—by the slide reduction algorithm of Gama and Nguyen [11]. They showed that slide reduction yields a Hermite exponent bounded by the Mordell constant $\mu _s$ and a modified approximation exponent (cf. (31)) bounded by $2\mu _s$,

32 $$\begin{aligned} h(g)\le \mu _s,~~~\widetilde{a}_{n-s+1}(g) \le 2\mu _s, \end{aligned}$$

appropriate since for a slide reduced basis *B*, the block $B^{n-s+1:n}$ is primal reduced. Similar, only slightly inferior results were proved by Li and Wei [19] when the maximal block size does not divide the dimension.

In this section we first discuss a new greedy LLL algorithm that is quasilinear in the bit sizes (when fast integer multiplication is used) and achieves the same guarantees for the shortest vector as all LLL algorithms. Previously, the only quasilinear time LLL algorithm were those of Novocin et al. [26] and Hanrot et al. [14], who obtained a provable constant bound for the Hermite exponent and (in [26]) for the approximation exponent.

We then introduce a simple way to analyze the self-dual SDBKZ variant of the BKZ reduction algorithm, recently introduced by Micciancio and Walter [21], improving on the dynamical system technique of Hanrot et al. [14]. We reprove their bound $\mu _s$ for the Hermite exponent [matching the first slide inequality in (32)] and prove a polynomial complexity result conjectured in [21]. The known techniques seem not sufficient to prove a bound for the approximation exponent of SDBKZ.

### LLL algorithms

An **LLL algorithm** is a block reduction algorithms that operates only on blocks of size 2. The acronym LLL refers to the initials of Lenstra, Lenstra and Lovász whose paper [18] contains the first such algorithm in arbitrary dimension and a proof of its polynomial complexity.

#### Proposition 3.1

Lagrange reduction of a block $B^{k:k+1}$ changes the bit profile to $g_i'$ in place of $g_i$ where $g_i'=g_i$ unless $i=k$. Moreover,

(i) $$\varepsilon :=g_k-g_k'$$ satisfies
  33 $$\begin{aligned} \Big (\frac{1}{2}c_k-\Gamma _2\Big )_+\le \varepsilon \le c_k, \end{aligned}$$
  where
  34 $$\begin{aligned} c_k:=2g_k-g_{k-1}-g_{k+1}=e_k-e_{k+1}. \end{aligned}$$
  and $a_+:=\max (a,0)$ denotes the positive part of a real number *a*.
(ii) For any $m, \displaystyle \max _{\ell \le m}e_\ell$ cannot increase and $\displaystyle \min _{\ell \le m}e_\ell$ cannot decrease. In particular, the $g_k$ remain bounded from below.

#### Proof

$\varepsilon \ge 0$ since a Lagrange step on the block $B^{k:k+1}$ cannot increase $g_k$. By Proposition 2.4 it reduces $c_k$ to $c_k'=c_k-2\varepsilon \le 2\Gamma _k$, giving the lower bound in (33). By Proposition 2.3, the new projected bit size is $e_k-\varepsilon =e_k'\ge e_{k+1}$, whence $\varepsilon \le e_k-e_{k+1}=2g_k-g_{k-1}-g_{k+1}=c_k$, giving the upper bound.

The first part of (ii) is an observation of Lenstra et al. [18, argument leading to (1.30)] that follows directly from (13). If $e:=\max e_\ell$ for the initial basis then this also holds for all later bases, and by induction, $g_k\ge g_n-(n-k)e$ for all *k*. Since $g_n$ remains invariant, we have bounded all $g_k$ from below. $\square$

A possible measure of the quality of a Lagrange reduction step is the amount $g_k-g_k'$ by which $g_k$ is reduced. If this is too small, there is no point performing the Lagrange reduction. Except for part (ii), the following bounds are proved along the lines of [18].

#### Theorem 3.1

Let $\delta >0$. If we accept a tentative Lagrange reduction step (performed at first only on the Gram matrix of the block) only when $g_k-g_k'>\delta$, an LLL reduction algorithm ends after finitely many successful Lagrange reduction steps.

(i) With $\Gamma _2^*:=\Gamma _2+\delta$, the final basis obtained satisfies
  35 $$\begin{aligned}&\displaystyle c_k\le 2\Gamma _2^* ~~~\text{ for } k=1,\ldots ,n-1, \end{aligned}$$
  36 $$\begin{aligned}&\displaystyle a_\ell -a_j\le 2\Gamma _2^*(\ell -j) ~~~\text{ for } \ell >j, \end{aligned}$$
  37 $$\begin{aligned}&\displaystyle h(g)\le a(g)\le c(g)=\frac{\sigma (g)}{n-1}\le 2\Gamma _2^*, \end{aligned}$$
  38 $$\begin{aligned}&\displaystyle A(g):=\sum _{k=1}^{n-1} g_k\le \frac{n-1}{2}g_n+{n+1 \atopwithdelims ()3}\Gamma _2^*. \end{aligned}$$
(ii) If the final basis has effective dimension $n_{{{\text{ eff }}}}=n$ then
  39 $$\begin{aligned} A(g)>\frac{n-1}{2}g_n-{n \atopwithdelims ()3}\frac{\Gamma _2^*}{2}. \end{aligned}$$
(iii) Given a basis whose components are integers of bit length at most $\beta$, an LLL algorithm performs
  40 $$\begin{aligned} N_{\text{ tot }}\le \delta ^{-1}n^2(\lg n+\beta ) \end{aligned}$$
  successful Lagrange reductions.

#### Proof

Proposition 3.1(ii) implies that $g_k$ can be reduced only finitely often by at least $\delta$. Thus the algorithm stops necessarily.

(i) By Proposition 3.1(i), if $c_k> 2\Gamma _2^*$ for some *k*, the gain in a Lagrange reduction at position *k* is $>\delta$, hence the reduction will be performed. Therefore no such *k* exists after convergence. This proves (35). (36) follows since
  $$\begin{aligned} a_\ell -a_j=e_j-e_\ell =c_j+c_{j+1}+\ldots +c_{\ell -1} \le 2\Gamma _2^*(\ell -j). \end{aligned}$$
  (37) now follows from (31), (30), and (8). Finally, one verifies
  $$\begin{aligned} A(g)=\frac{n-1}{2}g_n+\sum _{j=1}^{n-1}j(n-j)c_{j+1} \end{aligned}$$
  by substituting the definition of the $c_i$ into the sum and simplification. Since
  $$\begin{aligned} \sum _{j=1}^{\ell -1}j(\ell -j)={\ell +1\atopwithdelims ()3}, \end{aligned}$$
  this gives the bound (38).
(ii) If $n_{{{\text{ eff }}}}=n$ then $0<a_n=g_1+g_{n-1}-g_n=c_2+\ldots +c_n$, hence
  $$\begin{aligned} A(g)> & {} A(g)-\displaystyle \frac{n^2}{8}a_n=\frac{n-1}{2}g_n +\sum _{j=1}^{n-1}j(n-j)c_{j+1}-\frac{n^2}{8}\sum _{j=1}^{n-1}c_{j+1} \\= & {} \displaystyle \frac{n-1}{2}g_n-\frac{1}{8} \sum _{j=1}^{n-1}(n-2j)^2c_{j+1}\\\ge & {} \displaystyle \frac{n-1}{2}g_n- \sum _{j=1}^{n-1}(n-2j)^2\frac{\Gamma _2^*}{4}, \end{aligned}$$
  which gives (39).
(iii) Under the stated assumptions, the entries of *G* are bounded by $2^{2\beta }n$. The positive definiteness of *G* and Cramer’s rule therefore give
  41 $$\begin{aligned} 0\le g_k\le k\beta _k, \end{aligned}$$
  where
  42 $$\begin{aligned} \beta _k:=k^{-1}\lg k! + 2\beta +\lg n \le \lg (nk)+2\beta \end{aligned}$$
  since $k!\le k^k$. Since $g_k$ is nonnegative and decreases by at least $\delta$ with each reduction, it can be reduced at most $g_k/\delta$ times. Hence the total number $N_{\text{ tot }}$ of Lagrange reductions is bounded by
  $$\begin{aligned} N_{\text{ tot }}\le \delta ^{-1}\sum _{k=1}^{n-1} g_k \le \delta ^{-1}n^2(\lg n+\beta ) \end{aligned}$$
  since
  $$\begin{aligned} \sum _{k=1}^{n-1} \lg (nk)\le \int _{k=2}^{n-1} \lg (nk) dk<n^2\lg n,~~~ \sum _{k=1}^{n-1} k\beta ={n\atopwithdelims ()2}\beta <n^2\beta . \end{aligned}$$

$$\square $$

### Greedy LLL algorithms

To turn the general recipe into an efficient algorithm we must decide upon the order in which Lagrange steps are performed. Traditionally, these are chosen in a way determined by a fixed loop structure. In this section we consider greedy choices where in each step some utility measure is maximized. The measure in which we want to be greedy must be chosen carefully, in view of the following statement by Lovász on greediness in basis reduction: *”It seemed that the less greedy you were, the better it worked. So I only swapped neighboring vectors and only swapped when you really made progress by a constant factor.”* (Smeets [34, p.11]).

Storjohann [36, p. 13] suggested to perform each Lagrange step on the block $B^{k:k+1}$ for which the lower bound $\delta _k$ from (33) on the amount that $g_k$ decreases in a Lagrange reduction is largest. We shall call an algorithm that completes this description by a tie-breaking rule a **basic greedy LLL algorithm**. The basic greedy strategy can be observed experimentally to outperform many others. It was rediscovered by Zhao et al. [39] in the context of (low-dimensional) signal processing applications. Another greedy variant of LLL (and of slide reduction) was considered by Schnorr [31].

When $\beta$ is large and *n* is fixed, a basic greedy LLL algorithm typically performs only $O(1+\lg \beta )$ Lagrange reductions, which is much less than the bound (40). While a complexity bound of $O(1+\lg \beta )$ Lagrange reductions was proved by Hanrot et al. [14] for a **cyclic LLL algorithm** that performs Lagrange reductions on the blocks $B^{k:k+1}$ in increasing cyclic order, it seems to be impossible to prove for the basic greedy LLL algorithm an unconditional logarithmic complexity result. Schnorr [31] obtained only partial results, and had to assume an obscure technical condition with an early termination exit that endangers the quality of the reduced basis.

The main difficulty in the analysis is the possibility that the bit profile (which in the most typical cases has—apart from small randomly looking deviations—an essentially concave, nearly quadratic shape, reflected in a nearly monotone decreasing $e_i$ sequence) may exhibit large discontinuities. The top example of Fig. 1 illustrates such an atypical case from applications. Even more atypical cases arise when the effective dimension is less than the full dimension. Although one expects these cases to be reduced even more quickly than the regularly shaped ones, the tools presently available do not seem to allow one to demonstrate this.^2^

The technical obstacles can be overcome by changing the measure according to which the greedy choice is made.

A **special greedy LLL algorithm** applies Lagrange reductions always to blocks $B^{k:k+1}$ that maximize the scaling invariant number

43 $$\begin{aligned} \Delta _k:=\min \{c_k-2\Gamma _k,(k+1)g_k-kg_{k+1}\}, \end{aligned}$$

where $c_k$ is given by (34), until all $\Delta _k<\Delta$, where $\Delta >0$ is a small threshold. We may analyse the behavior of this greedy rule in terms of the **potential**

44 $$\begin{aligned} p:=\sum _{i=1}^{n-1} \Big ((k+1)g_k-kg_{k+1}\Big )_+. \end{aligned}$$

The proof of Theorem 3.1 shows that (38), which is the area under the bit profile, is a reasonable measure of how far a basis is from being reduced. If all terms in the sum (44) are positive then $p=2A(g)-(n-1)g_n$ is, up to a constant shift, twice this area; in general, *p* may be larger, accounting for an irregular behavior of the bit profile.

The potential is a convex, nonnegative function of the $g_i$. Therefore it attains its maximum at a vertex of any convex constraint set. Given only the dimension *n* and the maximal bit size $\beta$ of a basis with integral coefficients, the maximum potential with the constraints (41) is attained for a profile where all $g_i\in \{0,i\beta _i\}$. Writing $K:=\{i \mid g_i=0\ne g_{i-1}\}$ we find that the worst case for the potential has the form $p=\displaystyle \sum _{i\in K} 2i(i-1)\beta _{i-1}$. This is largest when $K=\{n,n-2,n-4,\ldots \}$, leading to an initial bound of

$$\begin{aligned} p^{{{\text{ init }}}}\le p^{\max } =\left\{ \!\! \begin{array}{cc} \displaystyle \sum _{j=1}^{n/2} 4j(2j-1)\beta _{2j-1} &{} (n\, \hbox {even})\\ \displaystyle \sum _{j=1}^{(n-1)/2} 4j(2j+1)\beta _{2j} &{} (n\, \hbox {odd})\end{array}\!\!\right\} \le \frac{n(n+2)(4n+1)}{3}(\beta +O(\lg n)). \end{aligned}$$

The following theorem shows that a special greedy LLL algorithm has a marginally better complexity than the cyclic LLL algorithm of Hanrot et al. [14], and at the same time gives stronger guarantees for the resulting reduced basis. (Hanrot et al. prove a constant bound on the Hermite exponent, but their method of analysis is unable to bound the approximation exponent.)

#### Theorem 3.2

Let

$$\begin{aligned} \Gamma _2^*:=\Gamma _2+\frac{1}{2}\Delta ,\,\, L_n:={n+1\atopwithdelims ()3},\,\,p^*:=2L_n\Gamma _2,\,\, q:=\frac{(p^{{{\text{ init }}}}-p^*)_+}{L_n\Delta }, \end{aligned}$$

where $p^{{{\text{ init }}}}$ is the potential of the input basis to a special greedy LLL algorithm. Then the algorithm stops after $N_{\text{ tot }}\le N_0$ Lagrange reductions, where

$$\begin{aligned} N_0:=\left\{ \begin{array}{ll} p^*/\Delta +1+L_n(1+\ln q) &{} if \, q>1,\\ p^{{{\text{ init }}}}/\Delta &{} otherwise.\end{array}\right. \end{aligned}$$

It returns a basis such that, for $1\le i\le k\le n-1$,

45 $$\begin{aligned}&\displaystyle c_k<2\Gamma _2^*\,\,\text{ or }\,\,(k+1)g_k-kg_{k+1} <\Delta , \end{aligned}$$

46 $$\begin{aligned}&\displaystyle \frac{g_i}{i}-\frac{g_k}{k}<(k-i)\Gamma _2^*, \end{aligned}$$

47 $$\begin{aligned}&\displaystyle a_{k+1}<2k\Gamma _2^*, \end{aligned}$$

48 $$\begin{aligned}&\displaystyle \max \Big \{2h(g),a(g),\frac{a_n}{n-1}\Big \}<2\Gamma _2^*. \end{aligned}$$

#### Proof

We put

49 $$\begin{aligned} p_i:= & {} (i+1)g_i-ig_{i+1},\nonumber \\ \Gamma := & {} \Gamma _2+\frac{1}{2}\max _i \Delta _i. \end{aligned}$$

Then the potential (44) takes the form

50 $$\begin{aligned} p=\sum _{i=1}^{n-1} (p_i)_+, \end{aligned}$$

and we have

51 $$\begin{aligned} \min (c_i-2\Gamma _2,p_i)=\Delta _i\le 2(\Gamma -\Gamma _2) ~~~\text{ for } i=1,\ldots ,n-1. \end{aligned}$$

Therefore (34) implies

52 $$\begin{aligned} p_i-p_{i-1}=ic_i\le i(2\Gamma _2+\Delta _i)\le 2i\Gamma , \end{aligned}$$

and we find by induction that

53 $$\begin{aligned} p_i\le i(i+1)\Gamma ~~~\text{ for } i=1,\ldots ,n-1. \end{aligned}$$

Summing these bounds for $p_i$ gives $p\le \displaystyle \frac{n^3-n}{3}\Gamma =2L_n\Gamma$. We conclude that at every iteration,

54 $$\begin{aligned} p-p^*\le 2L_n(\Gamma -\Gamma _2). \end{aligned}$$

By Proposition 3.1, Lagrange reduction of the block $B^{k:k+1}$ gives

$$\begin{aligned} p_k'=p_k-(k+1)\varepsilon ,~~~p_{k-1}'=p_{k-1}+(k-1)\varepsilon . \end{aligned}$$

The special greedy strategy guarantees that

55 $$\begin{aligned} 0\le \Delta _k=\max _i\Delta _i =2\Gamma -2\Gamma _2; \end{aligned}$$

in particular, $p_k\ge 0$ by (51). Therefore, the gain in the potential (50) is

$$\begin{aligned} p-p'=p_k+(p_{k-1})_+-(p_k')_+-(p_{k-1}')_+. \end{aligned}$$

To bound this from below we distinguish three cases.

**Case 1** Both $p_{k-1}'\le 0$ and $p_k'\le 0$. Then $p_{k-1}\le 0$, hence

$$\begin{aligned} p-p'=p_k. \end{aligned}$$

**Case 2** $p_{k-1}'> 0$ but $p_k'\le 0$. Then, using (52) and (33),

$$\begin{aligned} p-p'\ge p_k-p_{k-1}'=p_k-p_{k-1}-(k-1)\varepsilon \ge kc_k-(k-1)c_k=c_k >c_k-2\Gamma _k. \end{aligned}$$

**Case 3** $p_k'> 0$. Since $(p_{k-1}')_+\le (p_{k-1})_++(k-1)\varepsilon$, we find from (33) that

$$\begin{aligned} p-p'\ge p_k-p_k'-(k-1)\varepsilon =2\varepsilon \ge c_k-2\Gamma _k. \end{aligned}$$

This covers all cases, and we conclude from (51), (55), and (53) that always

$$\begin{aligned} p-p'\ge \min (c_k-2\Gamma _k,p_k)=\Delta _k=2\Gamma -2\Gamma _2 \ge L_n^{-1}(p-p^*). \end{aligned}$$

Therefore each Lagrange reduction produces a gain in the potential of at least $\Delta _k$, and we have

$$\begin{aligned} p'-p^*\le p-L_n^{-1}(p-p^*)-p^*=(1-1/L_n)(p-p^*)\le e^{-1/L_n}(p-p^*)_+. \end{aligned}$$

Now suppose first that $q>1$. Then after at most $L:=\lceil L_n\ln q\rceil \le 1+L_n\ln q$ Lagrange reductions,

$$\begin{aligned} (p-p^*)_+\le e^{-L/L_n}(p^{{{\text{ init }}}}-p^*)_+ \le q^{-1}(p^{{{\text{ init }}}}-p^*)_+ = L_n\Delta , \end{aligned}$$

hence $p\le L_n\Delta +p^*$. Therefore the algorithm stops after at most another $(L_n\Delta +p^*)/\Delta$ Lagrange reductions. It follows that the total number of Lagrange reductions is bounded by $p^*/\Delta +1+L_n(1+\ln q)$. On the other hand, if $q\le 1$ then there is essentially no geometric decay, and the algorithm stops after at most $p^{{{\text{ init }}}}/\Delta$ Lagrange reductions. This proves the complexity bound.

It remains to prove the guarantees (45)–(48) for the final basis. After termination, $\Delta _k<\Delta$ for all *k*, hence (55) implies

$$\begin{aligned} \Gamma =\Gamma _2+\frac{1}{2}\Delta _k<\Gamma _2+\frac{1}{2}\Delta =\Gamma _2^*. \end{aligned}$$

This implies (45) by definition (43). We may also rewrite inequality (53) as

56 $$\begin{aligned} \frac{g_k}{k}-\frac{g_{k+1}}{k+1}=\frac{p_k}{k(k+1)} \le \Gamma <\Gamma _2^*. \end{aligned}$$

Summing these gives (46). (47) follows from (46) since

$$\begin{aligned} a_{k+1}=g_1-\frac{g_k}{k}+\frac{p_k}{k}<(k-1)\Gamma _2^*+(k+1)\Gamma _2^* =2k\Gamma _2^*. \end{aligned}$$

Finally, (48) follows from the definitions, (47), and the particular case $i=1, k=n$ of (46), which gives

$$\begin{aligned} h(g)=\frac{1}{n-1}\Big (g_1-\frac{g_n}{n}\Big )<\Gamma _2^*. \end{aligned}$$

$$\square $$

For example, if we start with a basis in Hermite normal form then $g_1=\ldots =g_n=\beta$, hence $p_2=\ldots =p_n=\beta$, hence $p^{{{\text{ init }}}}=(n-1)\beta$, and we find $N_{\text{ tot }}=O(n^3\log (1+\beta /n^2))$.

A basis is LLL reduced in the traditional sense if the second alternative in (45) holds for all *k*. This is guaranteed by our theorem only when no final $p_k$ is tiny or negative. In view of (43), tiny or negative $p_k$ indicate a temporary barrier for reduction, which may or may not be lifted in later iterations. The final reduced basis is LLL reduced only in case all such barriers are ultimately lifted. However, the greedy LLL reduction guarantees for the most important key quality measures the same bounds (48) as a fully LLL reduced basis. (If needed, a fully LLL reduced basis can be obtained by continuing the LLL reduction as long as at least one of the reductions improves some $g_k$ by $>\frac{1}{2}\Delta$.)

If a basis *B* is greedy LLL reduced, the mean slope $a_n/(n-1)$ is bounded by the dimension-independent constant $2\Gamma _2=2-\lg 3\approx 0.415$ obtained from (48). For random reduced bases, the factor is better. A Lagrange reduced and size reduced Cholesky factor $$\left( \begin{array}{cc} r_1 &{} sr_1 \\ 0 &{} r_2\end{array}\right) $$ has $r_1^2/r_2^2\le 1/(1-s^2)$, hence $a_2=\lg (r_1^2/r_2^2)\le -\lg (1-s^2)$. Thus the expectation $\langle a_2\rangle$ of $a_2$ is bounded by

$$\begin{aligned} \langle a_2\rangle \le \overline{a}_2:=\langle -\lg (1-s^2)\rangle =-\langle \ln (1-s^2)\rangle /\ln 2. \end{aligned}$$

For example,

$$\begin{aligned} \overline{a}_2=\left\{ \begin{array}{ll}\Big (2-\ln \displaystyle \frac{27}{4}\Big )/\ln 2 \approx 0.1305 &{} \hbox {if}\,\, s\ \hbox {is uniformly distributed in}\ \Big [-\displaystyle \frac{1}{2},\frac{1}{2}\Big ],\\ \Big (1+3\ln \displaystyle \frac{3}{4}\Big )/\ln 2 \approx 0.1976 &{} \hbox {if}\,\, s^2\ \hbox {is uniformly distributed in} \ \Big [0,\displaystyle \frac{1}{4}\Big ].\end{array} \right. \end{aligned}$$

The empirical bound 0.16 for LLL-reduced bases of random lattices, calculated from remarks in Nguyen and Stehlé [24], is somewhere in between.

### SDBKZ reduction

In 2011, Hanrot et al. [14] introduced a variant of the BKZ algorithm of Schnorr and Euchner [32] that organized individual primal reduction steps into tours, in a way that the effect of a whole tour can be quantified. Hanrot et al. showed that exploiting the bit size inequalities introduced above reduces much of the complexity analysis to a study of linear equations and inequalities. Before their work, this underlying linear structure was invisible since the analysis was—with the single exception of Schönhage [33, Lemma 4.1]—always done in a multiplicative way.

Micciancio and Walter [21] use this technique to partially analyze a self-dual variant of the BKZ algorithm called SDBKZ. In this algorithm, given some block size *s* ($2<s<n$), tours of primal reduction of the blocks $B^{i:i+s-1}$ for $i=1,\ldots ,n-s$ and tours of dual reduction of the blocks $B^{i-s+2:i+1}$ for $i=n-1,n-2,,\ldots ,s$ alternate until no reduction gives an improvement.^3^ Assuming termination of the algorithm (which apparently happens in practice but was not demonstrated in theory) they proved for the resulting reduced basis the same bound $\mu _s$ on the Hermite exponent as one has for a slide reduced basis.

In the following, we simplify the complicated analysis of Hanrot et al. [14]. In particular, we present—as conjectured in [21]—a way to terminate the SDBKZ algorithm in polynomial time while essentially preserving the theoretical guarantees of the original SDBKZ. Moreover, the analysis suggests a way to skip certain reduction steps in the tours without compromising the quality of the output, thereby speeding up the algorithm.

Our analysis of the SDBKZ algorithm is based on a new global measure of the quality of a basis. As we saw in the analysis of the LLL algorithm, basis reduction amounts to shrinking the bit profile by making the bit sizes $g_i$ smaller. This can be done independently when the block size is $s=2$, which allows an elegant analysis of LLL algorithms. However, reducing one of the $g_i$ by primal or dual reduction of a block of size $s>2$ has an effect on some of the neighboring bit sizes that is not easy to quantify. One therefore needs to look for a suitable quality measure that has a predictable behavior under block primal or dual reduction.

The basic new idea is to consider the tightest parabolic dome that sits above the bit profile $g_0,\ldots ,g_n$ and interpolates the two end points. By setting up the interpolation conditions one finds that the curvature of the dome is characterized by the **bit defect**

57 $$\begin{aligned} \widetilde{\mu }:=\max _i\frac{1}{n-i}\Big (\frac{g_i}{i}-\frac{g_n}{n}\Big ). \end{aligned}$$

In particular, $\widetilde{\mu }$ is an upper bound for the Hermite exponent (24). When the bit defect is large, this dome is highly curved, and one expects to be able to gain a lot through reduction, while when the bit defect is tiny or even negative, this dome is flat or has a bowl shape, and only little can be gained.

One may now consider how the bit defect changes when one or more primal or dual reductions are applied to a basis. It turns out that this indeed works well for the cyclic BKZ algorithm analyzed in [14]; cf. the remarks further below. However, in order to apply the idea to the SDBKZ algorithm (which has the better theoretical bound on the Hermite exponent), we need to take into account that this algorithm does not perform any reductions of small blocks at the lower and upper end. For optimal results, one therefore needs to work with truncated versions of the bit defect, defined for a fixed block size $s>2$. The **primal bit defect**

$$\begin{aligned} \mu :=\max _{i\le n-s}\frac{1}{n-i}\Big (\frac{g_i}{i}-\frac{g_n}{n}\Big ), \end{aligned}$$

is the smallest number $\mu$ such that

58 $$\begin{aligned} \frac{g_i}{i}-\frac{g_n}{n}\le (n-i)\mu ~~~\text{ for } i=1,\ldots ,n-s. \end{aligned}$$

In particular, the case $i=1$ says that the Hermite exponent (24) satisfies $h(g)\le \mu$. If at some point $\mu \le \mu _s$, this implies the bound $\mu _s$ on the Hermite exponent guaranteed for slide reduction. Similarly, the **dual bit defect**

$$\begin{aligned} \mu ^\dagger :=\max _{i\ge s}\frac{1}{n-i}\Big (\frac{g_i}{i}-\frac{g_n}{n}\Big ), \end{aligned}$$

is the smallest number $\mu ^\dagger$ such that

59 $$\begin{aligned} \frac{g_i}{i}-\frac{g_n}{n}\le (n-i)\mu ^\dagger ~~~\text{ for } i=s,\ldots ,n. \end{aligned}$$

A small dual bit defect implies a good Hermite exponent of the dual basis.

The following theorem implies that, when started from an LLL reduced basis, the SDBKZ algorithm comes in polynomial time arbitrarily close to satisfying $\mu \le \mu _s$.

#### Theorem 3.3

Let $N_{\text{ tot }}(\mu ^*)$ be the number of (primal or dual) tours needed to reach (58) with $\mu \le \mu ^*$, when starting the SDBKZ algorithm with a dual tour. Then

$$\begin{aligned} N_{\text{ tot }}(\mu ^*)\le \Big \lceil N\log \displaystyle \frac{\mu ^{{{\text{ init }}}}}{\mu ^*-\mu _s}\Big \rceil ~~~\text{ for } \mu ^*>\mu _s, \end{aligned}$$

where

$$\begin{aligned} N:=\left\{ \begin{array}{ll}\displaystyle \frac{n-1}{s-1}&{} if\, n\le 2s+1,\\ \displaystyle \frac{n^2}{4s(s-1)} +1 &{} if\, n\ge 2s+2.\end{array}\right. \end{aligned}$$

#### Proof

We first note that Proposition 2.4 gives

60 $$\begin{aligned} g_i'-g_{i-1}-\frac{1}{s}(g_{i+s-1}-g_{i-1})\le \Gamma _s \end{aligned}$$

after primal reduction of the block $B^{i:i+s-1}$, and

61 $$\begin{aligned} g_i'-g_{i+1}-\frac{1}{s}(g_{i-s+1}-g_{i+1})\le \Gamma _s \end{aligned}$$

after dual reduction of the block $B^{i-s+2:i+1}$. We put

$$\begin{aligned} \mu ':=\mu _s+\Big (1-\frac{1}{N}\Big )(\mu -\mu _s) \le \mu _s+e^{-1/N}(\mu -\mu _s), \end{aligned}$$

and show by induction that if $\mu >\mu _s$ then at the end of the dual tour following the computation of $\mu$,

62 $$\begin{aligned} \frac{g_i'}{i}-\frac{g_n'}{n}\le (n-i)\mu ' ~~~\text{ for } i=s,\ldots ,n; \end{aligned}$$

i.e., the dual bit defect is now bounded by $\mu '$. Indeed, this holds trivially for $i=n$. Suppose that $i<n$ and (62) holds with $i+1$ in place of *i*. In step $i<n$ of the dual tour, $g_{i+1}$ has already been changed to $g_{i+1}'$. Noting that $g_n'=g_n$, (61) gives

$$\begin{aligned} g_i'\le & {} \Gamma _s+\Big (1-\displaystyle \frac{1}{s}\Big )g_{i+1}'+\frac{1}{s}g_{i-s+1} \\\le & {} (s-1)\mu _s +\Big (1-\displaystyle \frac{1}{s}\Big )(i+1)\Big ((n-1-i)\mu '+\frac{g_n}{n}\Big )\\&+\frac{i+1-s}{s}\Big ((n-1-i+s)\mu +\frac{g_n}{n}\Big ). \end{aligned}$$

We now put $\delta :=\mu -\mu _s>0$, substitute

$$\begin{aligned} \mu =\mu '+\frac{\delta }{N},\,\, \mu _s=\mu -\delta =\mu '+(1-N)\frac{\delta }{N}, \end{aligned}$$

and simplify to get

$$\begin{aligned} g_i'\le & {} i(n-i)\mu '+\displaystyle \frac{ig_n}{n} +\Big (\frac{(i+1-s)(n-1-i+s)}{s(s-1)}+1-N\Big )\frac{(s-1)\delta }{N}\\\le & {} i(n-i)\mu '+\displaystyle \frac{ig_n}{n} \end{aligned}$$

by choice of *N*. Thus (62) holds for *i*, and hence in general. If $\mu '\le \mu _s$, the goal is already achieved. Otherwise, we show that at the end of the subsequent primal tour we have

63 $$\begin{aligned} \frac{g_i''}{i}-\frac{g_n''}{n}\le (n-i)\mu '' ~~~\text{ for } i=1,\ldots ,n-s \end{aligned}$$

with

64 $$\begin{aligned} \mu '':=\mu _s+\Big (1-\frac{1}{N}\Big )(\mu '-\mu _s)\le \mu _s+e^{-2/N}(\mu -\mu _s); \end{aligned}$$

i.e., the primal bit defect is now bounded by $\mu ''$. Again, this is proved by induction. Since $g_0'=0$, (60) gives for $i=1$ the inequality

$$\begin{aligned} g_1''-\displaystyle \frac{g_n}{n}\le & {} \Gamma _s+\displaystyle \frac{g_s'}{s}-\frac{g_n}{n}\\\le & {} (s-1)\mu _s+(n-s)\mu '=(n-1)\mu _s+(n-s)(\mu '-\mu _s)\le (n-1)\mu '' \end{aligned}$$

since, as one easily shows, $N\ge \displaystyle \frac{n-1}{s-1}$. This proves (63) for $i=1$. Now suppose that (63) holds with $i-1$ in place of *i*. In step $i>1$ of the primal tour, $g_{i-1}'$ has already been changed to $g_{i-1}''$. Hence (60) gives

$$\begin{aligned} g_i''\le & {} \Gamma _s+\Big (1-\displaystyle \frac{1}{s}\Big )g_{i-1}''+\frac{1}{s}g_{i+s-1}' \\\le & {} (s-1)\mu _s +\Big (1-\displaystyle \frac{1}{s}\Big )(i-1)\Big ((n+1-i)\mu ''+\frac{g_n}{n}\Big )\\&\quad +\frac{i+s-1}{s}\Big ((n+1-i-s)\mu '+\frac{g_n}{n}\Big ). \end{aligned}$$

We now put $\delta ':=\mu '-\mu _s>0$, substitute

$$\begin{aligned} \mu '=\mu ''+\frac{\delta '}{N},~~~ \mu _s=\mu '-\delta '=\mu ''+(1-N)\frac{\delta '}{N}, \end{aligned}$$

and simplify to get

$$\begin{aligned} g_i''\le & {} i(n-i)\mu ''+\displaystyle \frac{ig_n}{n} +\Big (\frac{(i+s-1)(n+1-i-s)}{s(s-1)}+1-N\Big )\frac{(s-1)\delta '}{N} \\\le & {} i(n-i)\mu ''+\displaystyle \frac{ig_n}{n} \end{aligned}$$

by choice of *N*. Thus (63) holds for *i*, and hence in general.

As a consequence of (64), as long as the value of $\mu -\mu _s$ remains positive, it decreases every $\lceil Nt\rceil$ tours by a factor of at least $e^t$. This proves the theorem. $\square$

Without compromising the complexity order, one may run the algorithm in practice for up to $O(n^2)$ additional tours beyond those needed to reach $\mu \le \mu ^*$, where $\mu ^*$ is taken slightly larger than $\mu _s$. Since the apriori bound derived above is somewhat pessimistic for most (or even all?) lattice bases, a significantly smaller $\mu$ can typically be achieved. It is clear from the argument that only those reductions must be carried out for which $g_i$ does not yet satisfy the bound guaranteed by the above analysis. Thus only those reductions are carried out where $g_i$ is nearly largest when measured in the correct units. This introduces an element of laziness into the algorithm and speeds it up without affecting the worst case bound for the number of tours. For getting the first basis vector small quickly, it is also beneficial to begin the reduction with a dual rather than a primal tour. The reason is that a dual tour transports poor basis vectors towards higher indices and thus improves the quality of the leading blocks, which is then immediately exploited in the first step of the primal tour.

The cyclic variant of the BKZ algorithm analyzed in Hanrot et al. [14] proceeds by using primal tours only, but these are extended to shorter blocks towards the end of the basis. In this case, a similar analysis works, with the same *N* but using the symmetric bit defect defined by (57). The resulting new proof (whose details are left to the reader) is far simpler than that of [14] and results in the same convergence rate as given above for SDBKZ, which is a factor of approximately 16 better the bound on the rate derived in [14]. The final bound on $\mu$ and hence the Hermite factor resulting for BKZ is slightly weaker than that for SDBKZ.

Unfortunately, neither the above technique nor the original technique of Hanrot et al. is able to bound the approximation exponent or the enumeration exponent. In particular, unlike BKZ (where Schnorr [30] gives bounds on the approximation exponent) and slide reduction, SDBKZ is (at present) not guaranteed to find a very short vector in case that $\lambda _1(B)$ is much smaller than the trivial Hermite bound $\sqrt{\gamma _n d_n^{1/n}}$. (One could of course bound the approximation exponent by performing *O*(*n*) runs of SDBKZ according to the recipe of Lovász [20, p. 25].)

## Figures and Tables

**Table 2:**
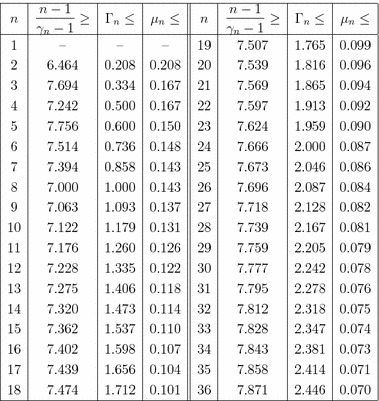
Best known upper bounds on Hermite constants

## Appendix: Hermite constants and Rankin invariants

The exact value of the Hermite constants $\gamma _n$ is known in dimensions $n\le 8$ [3] and $n=24$ [6]. Table 1 contains these values, the derived constants $\Gamma _n$ and $\mu _n$ from (11), and the corresponding extremal lattices, which, in these dimensions, happen to be unique up to isomorphism [38].

**Table 1:** Known exact Hermite constants

*n*	1	2	3	4	5	6	7	8	24
$\mathbb {L}$	$\mathbb {Z}$	$A_2$	$D_3$	$D_4$	$D_5$	$E_6$	$E_7$	$E_8$	$\Lambda _{24}$
$\gamma _n^n$	1	4 / 3	2	4	8	64 / 3	64	256	$2^{48}$
$\Gamma _n$	0	$(2-\lg 3)/2$	1 / 3	1 / 2	3 / 5	$(6-\lg 3)/6$	6 / 7	1	2
$\le$	0	0.2076	0.3334	0.5	0.6	0.7359	0.8573	1	2
$\mu _n$	−	$(2-\lg 3)/2$	1 / 6	1 / 6	3 / 20	$(6-\lg 3)/30$	1 / 7	1 / 7	2 / 23
$\le$	−	0.2076	0.1667	0.1667	0.15	0.1472	0.1429	0.1429	0.0870

The best asymptotic upper bound known is (according to Conway and Sloane [7, p.20])

$$\begin{aligned} \limsup _n \frac{\gamma _n-1}{n-1}=c<\frac{1}{9.793}. \end{aligned}$$

But this limiting value is approached very slowly. For small dimensions, better upper bound can be found using semidefinite programming techniques. Table 2 contains for dimension $n\le 36$ the best upper bounds known (apart from rounding), computed with correct directed rounding from the data in Cohn and Elkies [5]. Bounds valid for all dimensions are given in the following result.

### Theorem 3.4

(i) The Hermite constants satisfy the two-sided bounds
  65 $$\begin{aligned} \frac{1}{17.08}<\frac{1}{2e\pi }<\frac{\gamma _n-1}{n-1}\le \frac{1}{7} ~~~\text{ for } n>2; \end{aligned}$$
  moreover, $$\gamma _1=1$$ and $$\gamma _2=2/\sqrt{3}$$.

(ii) The upper bound $\frac{1}{7}$ in (65) is achieved precisely when $n=8$.

### Proof

The values for $\gamma _1$ is trivial and that for $\gamma _2$ is an old result by Lagrange [17]. The upper bound in (65) follows by combining the bound^4^

$$\begin{aligned} \gamma _n\le \frac{2}{\pi }\Gamma \Big (2+\frac{n}{2}\Big )^{2/n} =\frac{n}{e\pi }(1+o(1)) \end{aligned}$$

of Blichfeldt [2] with bounds for $n\le 36$ by Cohn and Elkies [5] (cf. Table 2). These bounds are strict unless $n=8$. (ii) follows from $\gamma _8=2$.

The lower bound in (65) follows from Ball [1]; note that $17.079<2e\pi <17.080$. $\square$

For Rankin invariants, Gama et al. [10] give the relation $\gamma _{ni}=\gamma _{n,n-i}$, the inequality

$$\begin{aligned} \gamma _{ni}\le \gamma _{nk}^{i/k}\gamma _{ki} ~~~\text{ for } i<k<n, \end{aligned}$$

and the special values $\gamma _{n1}=\gamma _n, \gamma _{nn}=1, \gamma _{42}=\frac{3}{2}$. Sawatani et al. [29] prove that $\gamma _{62}=3^{2/3}, \gamma _{82}=3, \gamma _{83}=\gamma _{84}=4$, and $2/\sqrt{3}\le \gamma _{63}\le \sqrt{6}, 2^{11/7}\le \gamma _{73}\le 2^{4/7}3^{2/3}$.

## Footnotes

1 Strictly speaking, the true bit sizes are the next largest integer of the present bit sizes. But the real-valued bit sizes introduced here are better adapted to the analysis.

2 Using *A*(*g*) as potential, one could proceed at first as in the proof of Theorem 3.2 below. However, the difficulty is to find, after the analogue of $p^*$ has been reached, a bound for the number of iterations that depends on *n* only, in order to preserve the logarithmic complexity in $\beta$ implicit in the $\log q$ term of Theorem 3.2. To get this bound one can use Theorem 3.1(ii)– but only when its hypothesis applies. Thus everything is ok with the basic greedy strategy if the effective dimension equals the full dimension. However, if the effective dimension decreases during the iteration, control is lost, and one has only the general complexity bound from Theorem 3.1, which is linear in $\beta$, not logarithmic. This is counterintuitive since in practice, a problem with a decreased effective dimension tends to take less work than a ”full” problem.

3 The original SDBKZ algorithm actually does primal reductions until $i=n-s+1$ and dual reductions until $i=1$, but this has no effect on the provable bounds.
